# Salt stress resilience in plants mediated through osmolyte accumulation and its crosstalk mechanism with phytohormones

**DOI:** 10.3389/fpls.2022.1006617

**Published:** 2022-09-26

**Authors:** Pooja Singh, Krishna Kumar Choudhary, Nivedita Chaudhary, Shweta Gupta, Mamatamayee Sahu, Boddu Tejaswini, Subrata Sarkar

**Affiliations:** ^1^Department of Botany, MMV, Banaras Hindu University, Varanasi, India; ^2^Department of Botany, School of Basic and Applied Sciences, Central University of Punjab, Bathinda, Punjab, India; ^3^Department of Environmental Science, School of Earth Sciences, Central University of Rajasthan, Ajmer, Rajasthan, India

**Keywords:** Brassinosteroids, ethylene, abscisic acid, cytokinins, Jasmonates, salicylic acids, osmolytes, salt stress

## Abstract

Salinity stress is one of the significant abiotic stresses that influence critical metabolic processes in the plant. Salinity stress limits plant growth and development by adversely affecting various physiological and biochemical processes. Enhanced generation of reactive oxygen species (ROS) induced *via* salinity stress subsequently alters macromolecules such as lipids, proteins, and nucleic acids, and thus constrains crop productivity. Due to which, a decreasing trend in cultivable land and a rising world population raises a question of global food security. In response to salt stress signals, plants adapt defensive mechanisms by orchestrating the synthesis, signaling, and regulation of various osmolytes and phytohormones. Under salinity stress, osmolytes have been investigated to stabilize the osmotic differences between the surrounding of cells and cytosol. They also help in the regulation of protein folding to facilitate protein functioning and stress signaling. Phytohormones play critical roles in eliciting a salinity stress adaptation response in plants. These responses enable the plants to acclimatize to adverse soil conditions. Phytohormones and osmolytes are helpful in minimizing salinity stress-related detrimental effects on plants. These phytohormones modulate the level of osmolytes through alteration in the gene expression pattern of key biosynthetic enzymes and antioxidative enzymes along with their role as signaling molecules. Thus, it becomes vital to understand the roles of these phytohormones on osmolyte accumulation and regulation to conclude the adaptive roles played by plants to avoid salinity stress.

## Introduction

Soil is a complicated system in which physical and biological processes interact. Various natural and anthropogenic activities influences climate change that leads to disturbed physical and chemical characteristics of soil (Pandey and Choudhary, 2019; Ahirwal et al., 2021). Among environmental changes, soil salinization has been known as a severe threat to agricultural fields (Mukhopadhyay et al., 2021). Approximately 20% of the world’s irrigated land [about 60 million (Mha)] has been globally affected due to salinity (FAO and ITPS, 2015), and with continuous climate change, it is projected to increase to 50% by 2050 (Machado and Serralheiro, 2017; Abdelrahman et al., 2018). In addition to this, FAO has reported that increasing soil salinity could pull 0.3–1.5 million hectares agricultural land out of production each year, reducing yield potential by 20–46 million hectares (FAO and ITPS, 2015). Therefore, crops cannot be cultivated if soil salinity is not controlled and rises above specified salinity thresholds (FAO, 2017). Among abiotic stresses, salinity affects plant growth by hampering photosynthesis, CO_2_ assimilation and excessive ROS production (Chaudhary and Choudhary, 2021; ElSayed et al., 2021; Selem et al., 2022). The detrimental effects of salinity begin with osmotic or water stress (a reduction in the root’s ability to absorb water), followed by ionic toxicity (nutritional imbalance, formation of ROS species), hormonal imbalance and susceptibility to infection by the pathogen (Choudhary et al., 2017; Talbi et al., 2018).

Plants respond to salinity by adapting diverse strategies such as phytohormonal regulation, redox change in potential, osmolyte biosynthesis, and epigenetic control of stress-related genes during stressful conditions. Similarly, salt stress tolerance is a complex trait that includes various signaling pathways, transcription factors, stress-responsive genes (Mansour and Hassan, 2022). However, tolerance to salinity levels varies between plant species, and such plants can be categorized as halophytes or glycophytes. Halophytes are the plants that are endowed with the ability to tolerate salinity up to 200 mM over glycophytes that are salt sensitive under adverse effects of salinity (Flowers and Colmer, 2008; Santos et al., 2016; Song et al., 2016). This salt tolerance mechanism in halophytes involves the reduction of Na^+^ influx, Na^+^ compartmentalization, and efflux of Na^+^ ions (Flowers and Colmer, 2008). In addition to this, ROS scavenging *via* antioxidant enzymes or quenching them with non-enzymatic molecules such as carotenoids, flavonoids, reduced glutathione, ascorbic acid and compatible osmolytes such as proline, glycine betaine (GB), trehalose sugar (Khan et al., 2015; Per et al., 2018) provide tolerance against salinity stress in the plant (Anjum et al., 2017). Osmolytes majorly contribute to maintaining cellular osmotic adjustment through cell turgidity, protects internal cell components and reduced ionic toxicity.

However, multiple pathways have been explored in the biosynthesis of osmolytes in bacteria as well as in plants. In addition to this, phytohormones undoubtedly regulate osmolyte production and accumulation (Per et al., 2017). Phytohormones interact synergistically with osmolytes and bring tolerance to stress (Iqbal et al., 2014). However, the comprehensive role of phytohormones by modulating the biosynthesis of osmolytes has not been explored properly. Therefore, it becomes imperative to understand the underlying mechanism of phytohormones regulating the biosynthesis of osmolytes. Thus, in this present review, we have focused on the synthesis and role of osmolytes in plants and further their modulation *via* phytohormones under salinity stress.

## Impact of salinity stress on plant growth

**Figure 1:**
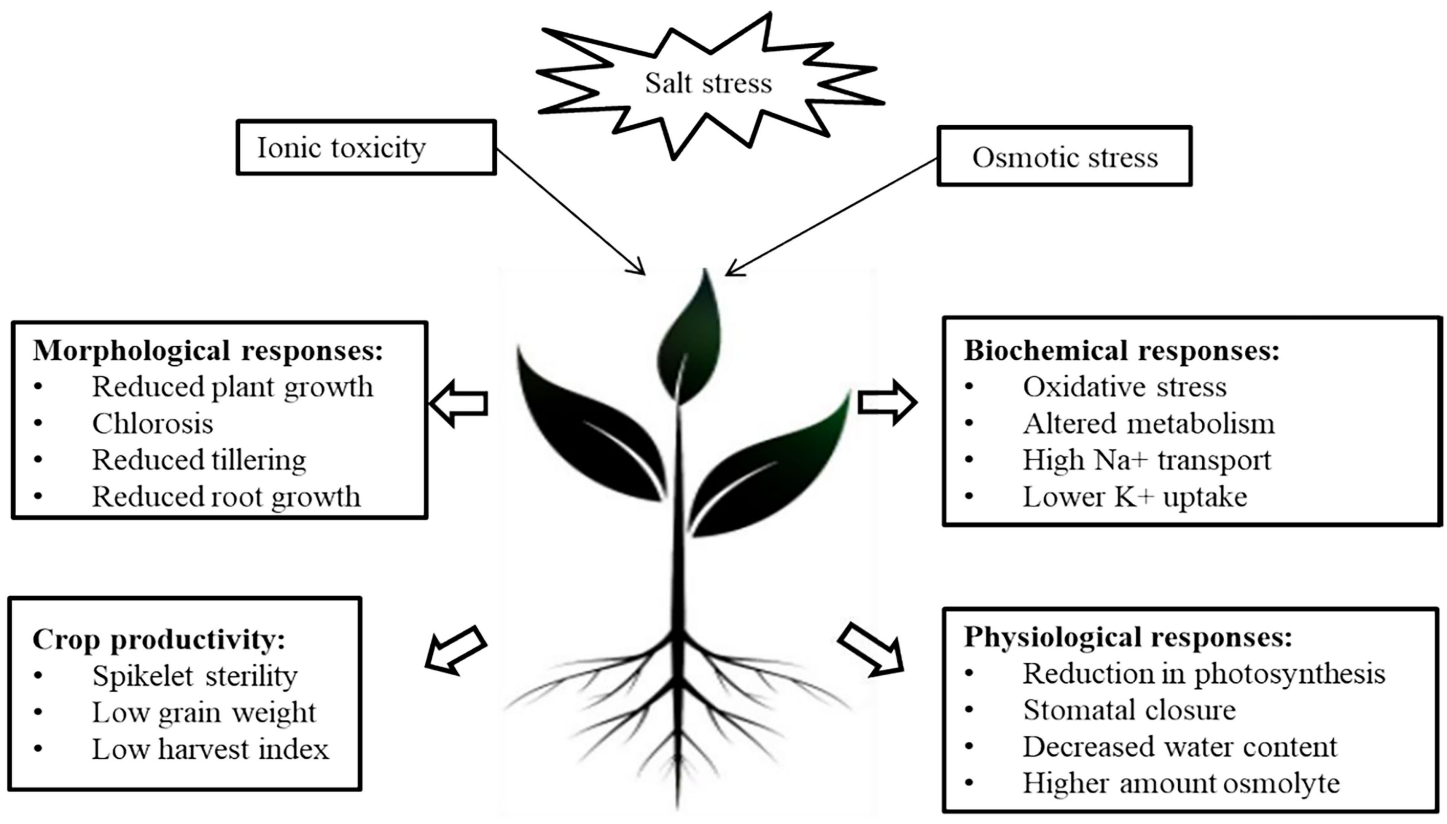
Impact of Salinity stress on various physiological and morphological traits of plants.

The term “Salinity” refers to the presence of an excessive amount of soluble salts in the soil that hinders plant growth (Etikala et al., 2021). High salinity is one of the major abiotic stress that is most widely distributed around the globe (Chaudhry and Sidhu, 2021). Since salinity is one of the stringent problems, it can be categorized as Primary and Secondary salinity. Primary salinity occurs in arid and semi-arid climatic zones due to natural, anthropogenic activities and Secondary salinity occur directly as a consequence of man-made activities (Safdar et al., 2019). The detrimental effect of salinity on crop growth is due to changes in physiological, morphological, biochemical and molecular responses in plant growth (Arif et al., 2020; Figure 1). Inhibitory effects of salt stress is influenced by number of factors including salt content, duration of exposure, plant species and varieties, photochemical quenching capability, plant growth stages, stress type, gas exchange characteristics, photosynthetic pigments, and ambient conditions (Shahverdi et al., 2018). At low levels of soil salinity, it enhances the plant length as concluded in various studies on various crops such as *Zea mays* (Hamada, 1995), *Oryza sativa* L. (Lee et al., 2011), *Vigna unguiculata* L. (Ibrahim, 2016), *Brassica campestris* L. (Memon et al., 2010) and *Vicia faba* L. (Hanafy et al., 2013). Higher sodium chloride salt concentrations, on the other hand, lowered the height of *Vigna mungo* L. (Kapoor and Srivastava, 2010), and *Tanacetum parthenium* L. (Mallahi et al., 2018) plants. Salt stress affects root and stem growth, and hinders nutrient uptake and translocation (Shrivastava and Kumar, 2015). Reduction in the plant growth is mainly due to decreased chlorophyll content which leads to the reduction in photosynthetic capacity of the plants under salinity stress (Netondo et al., 2004). In the context of plant growth, a recent detailed study on tomato with different salt concentrations (75,150 and 300 mM) exhibited a reduction in fresh and dry weight of roots (86.5% and 78.6%), shoot (71% and 72%), chlorophyll and carotenoid contents (22, 18.6%), and anthocyanin (41.1%), respectively (Alzahib et al., 2021). However, at a relatively higher concentration, 300 mM NaCl, reported an increase in proline content (67.37 mg g^-1^ fresh weight), antioxidant enzymes such as Superoxide dismutase (SOD), and Catalase (CAT), while reduction in malondialdehyde (MDA) content. An increased salt concentration alters the morphology, physiology and metabolism in these landraces in response to salt stress (Alzahib et al., 2021). Similarly, in *Medicago truncatula*, the effect of salinity on photosynthesis and chlorophyll fluorescence was studied, and total chlorophyll content was significantly reduced by 43% in TN6.18 and only 6% in TN.8.20 compared to control plants. The same effect was reported with carotenoid content, reduced by 51% in the sensitive TN6.18 line but only 13% in the resistant line (Najar et al., 2019). These recent studies indicate that salinity drastically affects the growth and development of plants. Nutritive imbalance another factor of salt stress that disrupts the osmotic equilibrium and further prevails drought conditions (Riaz et al., 2019). Additional effects include hampering reproductive processes such as inhibiting microsporogenesis, promotion of rapid programmed cell death and senescence of fertilized embryos (Suo et al., 2017). TEM micrographs of *Solanum melongena* treated with 75, 100, and 150 mM NaCl exhibited bulging chloroplasts and an absence of integrated thylakoid membranes associated with big starch grains (Alkhatib et al., 2021). Salinity influences the antioxidant activity of enzymes. For example, in *Andrographis paniculata* alteration in the activity of antioxidant enzymes, *viz.* catalase (CAT) and peroxidase (POD) was observed that further reveals the extent of induced changes modulated by salinity stress (Kumar and Srivastava, 2018). In short, the impact of salinity stress has been illustrated in Figure 1.

## Salinity stress tolerance in plants

To comprehend the physiological process of salinity tolerance in plants, one must first understand the cause of growth restriction, which can be due to salt’s osmotic impact on the soil or the toxic effect of salt within the plant body. As a result, plants respond to salinity in two ways: an initial rapid increase in external osmotic pressure at first followed by a gradual response as Na^+^ accumulates in leaves. In the first phase, plant roots sense the salt concentration above the threshold level, mainly 40 mM NaCl in many plants or less for sensitive plants like rice and *Arabidopsis*, and significantly decreases the rate of shoot growth. In the second ionic phase, the salt concentration increases excessively in the older leaves due to continuous transport to transpiring leaves over a longer period of time eventually results in higher salt concentration and then leaves die. As a result, if new leaves die faster than they are being produced, the plant’s photosynthetic capacity will no longer meet the carbohydrate requirements of young leaves, declining growth even more (Munns and Tester, 2008). Salinity tolerance is a physiological feature that is linked to a number of mechanisms that influences stress (Roy et al., 2014). However, based on the differential responses of plants, tolerance to salinity can be categorized into three types, *viz.* osmotic tolerance, ionic tolerance, and tissue tolerance (Chaudhary and Choudhary, 2021).

### Osmotic stress tolerance

Osmotic stress tolerance initiates a rapid response by diminishing cell expansion of root tips and young leaves and decreasing stomatal conductance to preserve water. It uses quick, long-distance (root to shoot) signaling pathways (Roy et al., 2014; Isayenkov and Maathuis, 2019), which essentially ignores the osmotic effects of NaCl, KCl, mannitol, and polyethylene glycol (Hassini et al., 2017; Darko et al., 2019; Prodjinoto et al., 2021; Gul et al., 2022). In osmotic tolerance, both organic solutes and inorganic ions are essential. These low molecular weight organic solutes, such as sugar and sugar derivatives including sucrose, polyols, and heterosides, GB and homarine (tertiary nitrogen compounds), amino acids such as proline, glutamate are commonly found in higher plants. Although it was thought that crop species had a wide range of osmotic tolerance, this was difficult to evaluate until recently. The measurements of growth parameters such as leaf growth and stomatal conductance methods are usually time-consuming and destructive. Digital images of plants allow measurements of the plant’s relative growth rate immediately after exposure to salinity and, hence, measure osmotic tolerance. Rice (Al-Tamimi et al., 2016), barley (Tilbrook et al., 2017), durum wheat (James et al., 2008; Sirault et al., 2009), bread wheat (Asif et al., 2018), and wild relatives of wheat, such as *T. monococcum,* have shown variations in osmotic tolerance based on relative plant growth rate.

### Ionic tolerance

**Figure 2:**
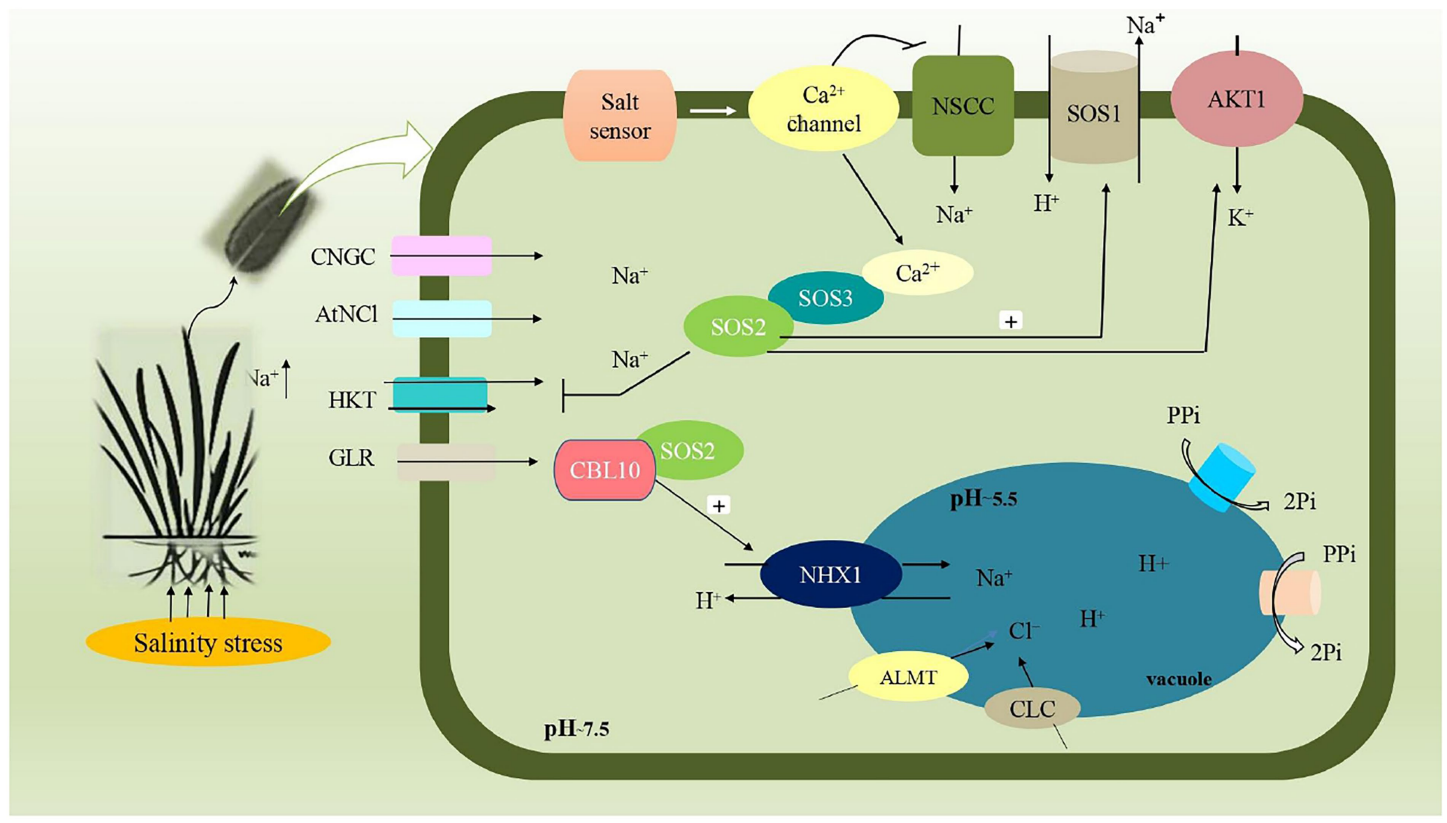
Schematic representation involved in salt overlay sensitive pathway (SOS) pathway during salinity stress. Plasma membrane of plant cell and organelle vacuolar membrane are involved in the transportation of Na^+^ ion. Apart from this, influx of Na^+^ into cells are mediated by ion transporters such as Cyclic-nucleotide gated channels (CNGs), Na^2+/^Ca^2+^ exchanger (AtNCl), High affinity K^+^ transporters (HKT), Glutamate receptors (GLR), Non-selective cation channels (NSCC). SOS1 (Na^+^/H^+^ antiporter), SOS2 (Serine/threonine protein kinase) and SOS3 (Calcium bindingprotein) are three genes commonly involved in this pathway that regulates the cytosolic concentration of Na^+^ ion, by subsequent extrusion and compartmentation into vacuoles. Calcineurin B-like (CBL10) along with SOS2 protein mediates the sequestration of Na^+^ ions into vacuole, through vacuolar membrane Na^+^/H^+^antiporter (NHX1), thereby maintaining ion homeostasis. Aluminum activated malate transporter (ALMT), Cation/chloride transporter (CLC) mediates the entry of Cl^−^ion into vacuole. Electrochemical potential is maintained by vacuolar H^+^-pyrophosphatase (AVP1) and H^+^-ATPase (V-ATPase).

The exclusion of ions, particularly Na^+^, from the shoot is a long-established mechanism for salinity tolerance in agricultural plants. This mechanism has received the most attention as it becomes easier to perform experimentation. Many crops, such as durum wheat (Forster, 2001; Munns and James, 2003), rice (Zhu et al., 2001; Lee et al., 2003), barley (Wei et al., 2003; Garthwaite et al., 2005) and *Medicago* (Sibole et al., 2003) have shown a substantial link between exclusion and tolerance of salt. In this mechanism, Na^+^ and Cl^−^ enter the plant’s roots and are quickly transported to the shoot *via* transpiration stream. To avoid the buildup of these ions into the shoot system, roots exclude most of the Na^+^ and Cl^−^ dissolved in the soil solution through which the concentration of salt in the shoot as a whole would never increase over that in soil, and the plant could survive indefinitely in saline soil. In this way, the concentration of Na^+^ and Cl^−^ ions is relatively higher in shoot than in roots that, improve the plant’s salt tolerance. Lauchli et al. (2008) evaluated the concentration of sodium and other ions in different layers of wheat root. In addition to this, the SOS1 antiporter localized to the root epidermis (particularly at the root tip, where roots are undifferentiated) provides the first line of defense against sodium uptake (Assaha et al., 2017; Figure 2).

### Tissue tolerance

Tissue tolerance refers to a tissue’s ability to retain tissue function while accumulating high amounts of intracellular Na^+^ or Cl^−^ ions (Munns et al., 2016; Negrāo et al., 2017). To avoid the detrimental effect of accumulated Na^+^ and Cl^−^ ions, compartmentalization into vacuoles or photosynthetically non-active cells avoids the accumulation of Na^+^ and Cl^−^ ions in the cytoplasm of plant cells where most important metabolic process occurs, i.e., tissue tolerance (Munns and Tester, 2008; Roy et al., 2014; Figure 2) and employing such mechanism will allow a plant to avoid toxicity. There is already a considerable amount of evidence across crop varieties in terms of the rates of accumulation of Na^+^ and Cl^−^ in the shoots (Munns et al., 2016).

## Major osmoprotectants in plants

Osmoprotectants are low molecular weight hydrophilic organic compounds that involve a variety of roles connected to plant defense mechanisms under varying environmental conditions. Unlike inorganic compounds, these compounds are non-toxic at higher cellular concentrations (Nahar et al., 2016; Niazian et al., 2021). During stressful conditions, these osmoprotectants accumulates in plants like proline, ectoine, trehalose, polyols, fructan, and quaternary ammonium compounds (QACs) such as glycinebetaine, alanine betaine, proline betaine, choline-*O*-sulfate, hydroxyproline betaine, and pipecolate betaine (Singh et al., 2015). Transgenic plants overexpressing biosynthetic enzymes for osmoprotectants, such as mannitol, GB, D-ononitol, or sorbitol, have accumulated these compounds in levels too low to give protective benefits solely through osmotic mass action (Huang et al., 2000).

The fundamental function of osmoprotectants accumulation in plants under salt stress is to maintain cell turgor pressure *via* osmoregulation, and protection of cellular components *via* reduction of ionic toxicity. Furthermore, by scavenging of hazardous ROS generated and preserving important antioxidative enzymes, these osmoprotective chemicals boost the antioxidative defense system in plants (Hasanuzzaman et al., 2014, 2019). In addition to this, osmolytes also function in the activation of defense-related genes under various stresses, which designates its prime importance in plants (Wani et al., 2018b). In the upcoming section, we discuss the role of important osmoprotectants under salinity stress in plants. Table 1 summarizes some of the relevant osmoprotectants in plants exposed to salinity stress.

**Table 1:** Biosynthetic pathways of osmoprotectants and their cellular functions.

Class	Osmoprotectants	Occurrence	Biosynthesis and Cellular functions	References
Amino acids	Proline (C_5_H_9_NO_2_)	Chloroplast and cytoplasm	Glutamate or ornithine pathway; acts as a molecular chaperone, maintains protein integrity and enzymatic activity of cellular enzymes, nitrogen storing agent, and ROS Scavenger	Verbruggen and Hermans (2008), Dar et al. (2016), and Ghosh et al. (2022)
	Alanine (C_3_H_7_NO_2_)	Cytoplasm	Glutamate is converted to pyruvate to form alanine by alanine aminotransferase; nitrogen storage under hypoxia conditions	Lea et al. (2007) and Miyashita et al. (2007)
	Arginine (C_6_H_14_N_4_O_2_)	Chloroplast	Synthesis of ornithine from glutamate to produce arginine; nitrogen storage and nitrogen immobilization during germination, the precursor for the biosynthesis of nitric oxide and polyamines	Slocum (2005), Winter et al. (2015) and Llebres et al. (2018)
	Glycine (C_2_H_5_NO_2_)	Chloroplast	Aspartate family pathway; signaling molecule in plant-gated glutamate receptors (GLRs)	Galili (2011) and Dubos et al. (2003)
	Glutamine (C_5_H_10_N_2_O_3_)	Chloroplast and cytosol	Catalytic condensation of glutamate and ammonia by enzyme glutamine synthetase; major amino acid donor for the synthesis of amino acids, and nitrogen-containing compounds; regulates gene expression of nitrate reductase, nitrate and ammonium transporter genes.	Kusano et al. (2011), Forde and Lea (2007), Vincentz et al. (1993), Sonoda et al. (2003), Vidmar et al. (2000), and Nazoa et al. (2003)
	Asparagine (C_4_H_8_N_2_O_3_)	Cytoplasm	Synthesis of asparagine and glutamate from aspartate and glutamine in ATP-dependent amino-transferase reaction catalyzed by asparagine synthetase; efficient molecule for nitrogen storage and transport.	Lomelino et al. (2017) and Lea et al. (2007)
	γ-Amino-Butyric Acid (C_4_H_9_NO_2_)	Cytosol	Irreversible decarboxylation of glutamate catalyzed by glutamate decarboxylase; acts as signaling molecule in plant growth and development; stomatal regulation; free-radical Scavenging activity	Li et al. (2021)
Sugars	Trehalose (α-D-glucopyranosyl-1-α-D glucopyranoside) (C_12_H_22_O_11_)	Cytoplasm	Two-step process involving trehalose-6-phosphate (T6P) production catalyzed by trehalose-6-phosphate synthase (TPS) and its subsequent dephosphorylation to trehalose mediated by trehalose-6-phosphate phosphatase (TPP); non-reducing sugar, protects cellular membranes and proteins by formation of amorphous glass structure	Ponnu et al. (2011), Abdallah et al. (2016) and Kosar et al. (2018)
	Sucrose (a-D-glucopyranosyl-β-Dfructofuranoside) (C_12_H_22_O_11_)	Cytosol	UDP-glucose & fructose-6-phosphate are converted into sucrose-6-phosphate by SPS and finally to sucrose by SPP; act as both metabolite and signaling molecule in plant metabolism and development	Wind et al. (2010), Tognetti et al. (2013), and Ruan (2014)
	Fructose (C_6_H_12_O_6_)	Cytosol	Sucrose is converted into fructose *via* INV; protects membranes or other cellular components as fructans, directly influences plant growth.	Dennis and Blakeley (2000)
	Maltose (C_12_H_22_O_11_)	chloroplast	Hydrolysis of α-1,4 glycosidic linkage of polyglucan chains to produce maltose; protects membrane proteins and photosynthetic electron transport chain.	Kaplan and Guy (2004) and Kaplan et al. (2006)
	Galactinol (1-O-alpha-D-galactopyranosyl-L-myo-inositol) (C_12_H_26_O_13_)	Cytoplasm	UDP-galactose and myo-inositol synthesizes GOl with key enzyme galactinol synthase (GOlS);RFO biosynthesis protects the plant against biotic and abiotic stress.	dos Santos and Vieira (2020)
Quaternary Ammonium Compounds	Glycine-Betaine (N,N,N-trimethyl glycine) (C_5_H_12_NO)	chloroplast	Choline is converted to betaine aldehyde and then to glycine betaine through CMO; protects membrane enzymes and proteins, and safeguards young leaves and tissues during the onset of stress.	Annunziata et al. (2019)
	*β*-Alanine Betaine (C_6_H_13_NO_2_)	Cytoplasm	*S*-adenosylmethionine-dependent *N-*methylation of beta-alanine *via N-*methyl beta-alanine and *N,N-*dimethyl beta-alanine; appropriate osmoprotectant than glycine betaine	Parthasarathy et al. (2019)
	Proline betaine (*N,N-*dimethylproline or stachydrine) (C_7_H_13_NO_2_)	Chlorophyll containing tissue	Synthesized from several steps of methylation of proline under long-term response to salinization; more effective osmoprotectant than proline in bacteria	Trinchant et al. (2004)
	Choline*-O-*Sulfate (C_5_H_13_NO_4_S)	Chloroplast	Choline and 3′-phosphoadenosine 5′-phosphosulfate (PAPS); detoxification activity and osmoprotection	Hanson et al. (1994) and Hagihara et al. (2012)
	TMAO (Trimethylamine N-oxide) (CH_3_)_3_NO	-	Synthesized from plant FMOs; enhances protein folding in plants and up-regulates the abiotic stress-induced gene expression	Catala et al. (2021)
Sugar Alcohol	D-pinitol (C_7_H_14_O_6_)	Cytoplasm	Conversion of glucose phosphate precursor to myo-inositol by the action of INPS (myo-inositol 1-phosphate synthase) and IMP (myo-inositol monophosphatase) which is methylated. and epimerized to form D-pinitol in a two-step reaction process; able to maintain turgor pressure that in turn confers osmotic adjustment.	Ahn et al. (2018) and Dumschott et al. (2019)
	Mannitol (C_6_H_14_O_6_)	Cytoplasm	Enzymatic action of mannose-6-phosphate isomerase, mannose-6-reductase and mannose-1-phosphate phosphatase on fructose-6-phosphate; osmotic adjustment, regulation of redox system (ROS Scavengers), molecular chaperons	Loescher et al. (1992) and Kaya et al. (2013)
	Myo-Inositol (C_6_H_12_0_6_)	Cytoplasm	*De novo* biosynthesis of myo-inositol from D-Glucose-6-phosphate catalyzed by MIPS (myo-inositol 1-P synthase) followed by dephosphorylation by inositol monophosphatase; membrane biogenesis, phosphorus storage, secondary messenger, osmotolerance	Majumder et al. (2003) and Dastidar et al. (2006)
	Sorbitol (C_6_H_14_O_6_)	Cytoplasm	Action of sorbitol-6-phosphate dehydrogenase (S6PDH) and sorbitol-6-pyrophosphatase (S6PP) on glucose-6-phosphate to produce sorbitol; osmoprotectant and major photosynthetic product and catalyze the oxidation of sorbitol to fructose.	Dutta et al. (2019)
	D-ononitol (C_7_H_14_O_6_)	Cytoplasm	Myo-inositol can be methylated by myo-inositol-O-methyltransferase (IMT) to form D-ononitol; prevent water loss in plants, thus providing salt and drought tolerance	Streeter (1985) and Handa et al. (2018)
Polyamines	Putrescine (Put) (C_4_H_12_N_2_)	Cytosol	Ornithine or arginine *via* decarboxylation reactions; maintain cellular pH and ionic balance, osmotic adjustment, hydroxyl radical scavengers, down-regulate methylglyoxal production.	Gill and Tuteja (2010a,b), Kuznetsov et al. (2007) and Pál et al. (2021)
	Spermidine (Spd) (C_7_H_19_N_3_)	Cytosol	From putrescine *via* spermidine synthase, quench singlet oxygen, osmoprotectant, enhances plant growth through reactive oxygen metabolism.	Gill and Tuteja (2010a,b), Meng et al. (2015), Baniasadi et al. (2018), and Pál et al. (2021)
	Spermine (Spm) (C_10_H_26_N_4_)	Cytosol	From spermidine *via* spermine synthase; enhances the accumulation of ABA maintaining cellular homeostasis.	Gill and Tuteja (2010a,b), Marco et al. (2019), and Pál et al. (2021)

ABA, abscisic acid; FMOs, Flavin-containing monooxygenases; GABA, γ-aminobutyric acid; GOl, Galactinol; INV, invertase; RFO, Raffinose family of Oligosaccharides; ROS, reactive oxygen species; SPS, Sucrose phosphate synthase; SPP, Sucrose-phosphate phosphatase; UDP-glucose, Uridine diphosphate glucose.

### Amino acids

Amino acids derived osmoprotectants such as proline, arginine, alanine, leucine, glycine, serine, valine and γ-aminobutyric acid (GABA; Suprasanna et al., 2014). These osmoprotectants accumulate under salinity stress conditions and decrease the osmotic potential of cells allowing water absorption. They also stabilize protein structures and membranes (Ashraf and Foolad, 2007); also act as nitrogen storing agents and ROS scavengers (Hayat et al., 2012). In stressed conditions, accumulation of mainly proline in relatively higher amounts as compared to other osmoprotectants is an indication of stress conditions. *β*-alanine being a non-proteinogenic amino acid is a stress response molecule involved in plant protection from biotic and abiotic stresses. Furthermore, it is converted into *β*-alanine betaine, an osmoprotective compound in some plant species (Parthasarathy et al., 2019).

#### Proline

Proline can be biosynthesized by two pathways: glutamate and ornithine pathway. Proline is produced from glutamatic acid *via* the intermediate Δ^1^-pyrroline-5-carboxylate (P5C), which is catalyzed by Δ^1^-pyrroline 5-carboxylate synthetase (P5CS) and Δ^1^-pyrroline5-carboxylate reductase (P5CR) in the glutamate pathway (Dar et al., 2016). However, in an alternate pathway, Proline is synthesized from ornithine (Orn), which is transaminated to Pyrroline-5- carboxylate (P5C) *via* Orn-*δ*-aminotransferase (*δ*-OAT; Verbruggen and Hermans, 2008). It has been claimed that proline buildup aids stress tolerance in various ways. It acts as a molecular chaperone, ensuring the integrity of proteins and enhancing enzyme activity (Ghosh et al., 2022). Recently, engineered plants have high expression of pyrroline-5-carboxylate reductase enzyme, leads to the accumulation of proline. Also, it has been observed that the antioxidant property of proline is helpful as ROS Scavenger (El-Badri et al., 2021). The importance of the Orn pathway in the development of rice seedlings has been explained through the constitutive expression of OsOAT genes. These genes are responsible for enhanced *δ*-OAT activity, improved antioxidant status, tolerance to drought, and osmotic stress (You et al., 2012). However, under salt stress, the preferential use of the Glu pathway over the Orn pathway is increased due to enhanced expression of P5CS activity. This suggests the pivotal role of Glu pathway in proline accumulation during osmotic adjustment (Zhen and Ma, 2009). Delauney et al. (1993) reported that *P5CS* mRNA levels were significantly up-regulated while OAT mRNA levels were down-regulated during salt stress in *Vigna aconitifolia* L. Later, it was confirmed by Lei et al. (2016) and Mansour and Ali (2017) through evaluation studies related to proline biosynthesis in salt stress. Interestingly, exogenous application of proline produced different results. For instance, *Zea mays* L. under foliar exposure of proline resulted in decreased P5CS activity and increased PDH activity under salt stress (de Freitas et al., 2018). Similar results were obtained in *Sorghum bicolor* under saline conditions (de Freitas et al., 2019). Seed primed with exogenous proline has been observed with decreased *P5CS* response while, on the other hand, PDH expression increased significantly in *Triticum aestivum* L. (Rady et al., 2019). These increased PDH level protect plants from proline toxicity (Deuschle et al., 2001). Over-expression of *P5CS* gene expression in *Lepidium draba* leads to proline accumulation and improved antioxidative responses under salt stress (Pakzad et al., 2021).

### Quaternary ammonium compounds

Quaternary ammonium compounds like GB, β-alanine betaine, proline-betaine, choline-*O*-sulfate etc. (Koyro et al., 2012) accumulate under salt stress conditions. Among these compounds, GB generally gets accumulated in a more considerable amount as compared to others, mainly accumulated in the chloroplast. GB helps to maintain intracellular osmotic equilibrium by regulating water flow into the cells (Ranganayakulu et al., 2013). More benefits are reflected in terms of the protection to thylakoids membrane, maintenance of photosynthetic activity and stomatal conductance, and photorespiration reduction etc. Transgenic approaches by overexpressing Betaine aldehyde dehydrogenase (BADH) enzyme provide better tolerance under stressful conditions (Sulpice et al., 2003).

#### Glycine betaine

Glycine betaine (GB) is zwitterionic, neutral at physiological pH and a quaternary ammonium compound that is N-methylated derivative of glycine (Ashraf and Foolad, 2007). GB is widely synthesized in chloroplasts of young tissues protecting the membrane enzymes and proteins under stressful environmental conditions. Because GB is not actively destroyed or metabolized in plant tissues, its concentration is determined by synthesis, transport, and dilution of plants (Annunziata et al., 2019). GB is biosynthesized as spatio-temporal under abiotic stress conditions (Annunziata et al., 2019). Usually, it is synthesized at modest levels and gently rises in young tissues /organs when abiotic stress occurs. Furthermore, unlike proline, the GB is swiftly re-translocated to younger leaves even if it is exogenously supplied to older sections. Thus, it can be inferred that GB cannot be metabolized and plays a critical role in the protection of young tissues.

Under normal and stressful conditions, some plant species accumulate GB spontaneously (Chen and Murata, 2008) such as major cereals that are completely devoid of the potential to accumulate GB (Kurepin et al., 2015). An attempt to transfer GB biosynthetic genes in these plants *via* genetic engineering is considered a protective strategy for increasing salinity stress tolerance (Chen and Murata, 2011). Upregulation of the BADH gene has been reported as a potential biomarker in salt-stressed wheat plants (Lv et al., 2016). Similarly, *Arabidopsis* plants transformed with a novel BADH gene, *ScBADH,* resulted in the accumulation of SOD, proline and GB under salt stress (Wang et al., 2016). In another study, transgenic maize introduced with BADH gene from *Artiplex micrantha* L. reported higher GB content that is sufficient to impart tolerance against salt stress.

Similarly, japonica and rice cultivars mediate up-regulated expression of the BADH1 gene in salt stress (Fitzgerald et al., 2008). Thus, it could be inferred that BADH act as a positive regulator in the treatment of salt stress *via* MAPK pathway in plants. Furthermore, the codA gene (choline oxidase) isolated from *Arthrobacter globiformis* can potentially alleviate phosphate deficiency in tomato plants through GB action (Li et al., 2019). In addition, GB regulates ion channels and transporters by maintaining Na^+^/K^+^ levels that are helpful in the transportation of phosphate under salt-stressed conditions (Haruta and Sussman, 2012; Wei et al., 2017; Yuan et al., 2017).

#### Trimethylamine N-oxide

Plant osmolytes, besides enhancing osmotic homeostasis and stabilization, also act as chaperons in preserving the folding of proteins under stressful conditions and thus providing stability and function (Slama et al., 2015; Rabbani and Choi, 2018). In animals, TMAO is a quintessential osmolyte that act as a chaperone that retains the folding status of protein and protects against denaturants (Yancey, 2005; Strambini and Gonnelli, 2008; Jethva and Udgaonkar, 2018). Interestingly, a recent study demonstrated that the plant also synthesizes TMAO endogenously from FAOs and further illustrates that its level rises under abiotic stress conditions. Thus, TMAO enhances plant tolerance to freezing, drought, temperatures and high salt (Catala et al., 2021).

### Sugars

During salt stress, carbohydrates such as sugars (e.g., glucose, fructose, trehalose) and starch accumulate (Parida et al., 2004). The significant role played by these sugars involves osmoprotectant and scavenging of ROS for mitigating salt stress. It has also been reported that with an increase in the level of salt stress, the level of reducing sugars (sucrose, fructans) also increases significantly (Kerepesi and Galiba, 2000; Gangola and Ramadoss, 2018).

#### Trehalose sugar

Trehalose (Tre) is a non-reducing sugar that is made up of two glucose residues (D-glucopyranose units) linked together by an extremely stable linkage (Ahluwalia, 2022). It’s a suitable solute or osmoprotectant even at high cellular concentrations because it’s non-reducing in nature and very soluble (Lunn et al., 2014).

Plants produce Tre through the trehalose-6-phosphate synthase/phosphatase (*OtsA*–*OtsB*) pathway, which is dependent on two essential molecules: uridine-diphospho-glucose (UDP-Glc) and glucose-6-phosphate (Glc-6-P) in a two-reaction process (Paul et al., 2008; Kosar et al., 2018). Tre functions as a protective molecule for cellular, membrane, and proteinaceous structures due to this particular characteristic (López-Gómez and Lluch, 2012; Abdallah et al., 2016). Tre preserves membrane and protein structures under stress by forming an amorphous glass structure and influencing the surrounding polar phospholipid head groups or amino acids *via* hydrogen bonding (Einfalt et al., 2013; Poonam et al., 2016). The creation of this amorphous glassy structure protects biomolecules from the negative effects of abiotic stresses, particularly dehydration and aids in the recovery of their specific functions when normal non-stress environmental conditions prevail (Kosar et al., 2018). Transgenic plants have conferred enhanced tolerance to various abiotic stresses through the expression of Tre biosynthesis genes (Iordachescu and Imai, 2008). TPP (trehalose-6-phosphate phosphatase) and TSS (trehalose-6-phosphate synthase) genes are commonly present in the genomes of higher plants (Leyman et al., 2001). *OsTPP1*, a member of the TPP gene family, is involved in circumventing salt tolerance through transient up-regulated expression (Chao et al., 2005). For instance, the overexpressed *OsTPP1* gene in rice plants has been found to tolerate salt stress by promoting the expression of stress-responsive genes (Ge et al., 2008). Similarly, Tre accumulation takes place in transgenic rice plants through regulated expression of fusion genes involving *E. coli* biosynthetic genes (*OtsA* and *OtsB*) along with TPS and TPP genes (Garg et al., 2002). These plants exhibited high tolerance levels to salt stress, further necessitating the importance of these genes in the design of transgenic plants. In *Arabidopsis*, AtTPPD is a chloroplast-localized enzyme that has been speculated to enhance tolerance toward high salinity (Krasensky et al., 2014). Higher accumulation of soluble sugars and starch levels achieved through over-expression of AtTPPD suggested its putative role in the metabolism of sugars under salt stress. The introduction of TPP gene from rice into maize plants resulted in a 20%–31% higher yield than non-transgenic controls (Nuccio et al., 2015).

### Sugar alcohol

Sugar alcohols such as pinitol, mannitol, myo-inositol and sorbitol show an influential role in mitigating stress conditions by adjusting osmotic equilibrium. These are also well-known as polyols. Myo-inositol and Pinitol have a cyclic structure, while mannitol and sorbitol have a linear structure. Their accumulation in plants is thought to serve a variety of functions, including osmotic adjustment, ROS regulation and molecular chaperons (Upadhyay et al., 2015; Bhattacharya and Kundu, 2020). Mannitol, a sugar alcohol, formed by the action of enzyme mannose-6-phosphate reductase on the mixture of glucose/fructose transforms into mannitol and gluconic acid. Mannitol aids in osmotic regulation and helps to remove oxygen radicals produced by stress (Kaya et al., 2013). Sorbitol is an osmoprotectant that is produced during photosynthesis (Wu et al., 2010). Zhou et al. (2003) utilized sorbitol-6-phosphate to produce sorbitol by dephosphorylating sorbitol-6-phosphatase. D-Ononitol, on the other hand, is a sugar alcohol that acts as an osmolyte, reducing water loss in plants during drought stress. The myo-inositol O-methyltransferase gene from *Mesembryanthemum crystallinum* was transferred into tobacco, which resulted in increased D-ononitol production and improved drought and salt resistance (Vinocur and Altman, 2005). Pinitol is generated by the methylation of myo-inositol and has found in many halophytic species. Ononitol epimerization also results in the formation of pinitol (Sengupta et al., 2008; Slama et al., 2015; Dumschott et al., 2019).

#### Mannitol

Mannitol is an acyclic polyol consisting of six carbon atoms (Slama et al., 2015). It is essential in quenching hydroxyl radicals (Gill and Tuteja, 2010a,b). Mannose-6-P isomerase (phosphomannose isomerase), mannose-6-phosphate reductase, and mannose-1-phosphate phosphatase are among the enzymes that start the production of mannitol in plants from Fructose-6-P (Loescher et al., 1992). As mannitol accumulates spontaneously in all plant species, adding it to non-mannitol accumulators can improve their resistance to adverse environmental conditions. Similarly, the incorporation of mannitol biosynthetic genes from accumulator species to non-accumulator species enhances their resistance to abiotic challenges, which is also a successful strategy for mitigating adverse impact of climate change. Eggplants engineered with *mtlD* gene have established tolerance against high NaCl stress (200 mM) (Prabhavathi et al., 2002). *Populus tomentosa, a* transgenic woody plant transformed with the *mtlD* gene encoding mannitol-1-phosphate dehydrogenase, has survived salt stress as a result of mannitol’s oxidative stress protection (Hu et al., 2005). This *E. coli* related *mtlD* gene encodes non-specific phosphatases that convert mannitol-1-phosphate to mannitol in transgenic plants.

Further, mannitol-synthesizing transgenic peanut plants resulted in the accumulation of mannitol by over-expression of the *mtlD* gene from *E. coli* under salt stress (Bhauso et al., 2014). Moreover, the antioxidant genes might influence the expression of the *mtlD* gene in maintaining cellular homeostasis through detoxification of ROS species during salt stress (Patel et al., 2016). Recently, an attempt to the production of mannitol synthesis in *Arabidopsis thaliana* has been reported by expression of two biosynthetic genes, namely, mannitol-1-phosphate dehydrogenase and mannitol-1-phosphatase genes from brown algae *Ectocarpus* sp. strain Ec32, that results in the production of 42.3–52.7 nmol g^−1^ fresh weight of mannitol, sufficient to impart salinity stress tolerance (Rathor et al., 2020).

#### Inositol

Inositol or more preciously myo-inositol is a sugar-like carbohydrate produced in many plants (Valluru and Van den Ende, 2011; Nisa et al., 2016). Being an osmoprotectant, its derivatives including pinitol, galactinol and ononitol, also perform diverse functions as osmoprotectant (Handa et al., 2018). It also regulates the synthesis of phytohormone such as auxin, phytic acid biosynthesis, and plant defense mechanism (Hazra et al., 2019). Inositol and other related molecules are suggested to prove salt tolerance in two ways: (1) protection of cellular structures from ROS, (2) maintaining cell turgor pressure inside the cell. The production of inositol is a two-step metabolic route that begins with the enzymatic conversion of d-glucose 6-P into myo-inositol-1-P catalyzed by myo-inositol-1-P synthase (Majumder et al., 1997), followed by dephosphorylation of myo-inositol-1-P resulting to form myo-inositol that further produces different inositol-containing compounds such as phospholipids (Dastidar et al., 2006). Introgression of salt tolerant MIPS (L-myo-inositol 1-phospahte synthase) protein encoded by *PcINO1* gene of *Porteresia coarctata* resulted in inositol accumulation in tobacco plants (Majee et al., 2004). Transcriptome analysis studies revealed the potential of over-expression of the *IbMIPS1* gene in transgenic sweet potato plants induced *via* salt stress (Zhai et al., 2016). Another *MIPS* gene, *MdMIPS1 (myo-inositol-1-phosphate synthase1)* overexpressed in transgenic apple plants, has promoted the biosynthesis of myo-inositol along with the accumulation of other osmoprotectants to alleviate salinity-induced osmotic stress (Hu et al., 2020).

### Polyamines

Polyamines (PAs) are nitrogen-containing compounds having a low molecular weight that are present in cellular compartments. The most common PAs found in plants are spermidine (Spd), putrescine (Put), and spermine (Spm), classified as plant growth substances (Bano et al., 2020). Several other PAs such as cadaverine, homospermidine, canavalamine, and 1, 3-diamino propane are synthesized from amino acids. PAs generally occur in free or conjugated form with macromolecules or phenolic compounds. Put, Spd, and Spm are the most abundant PA that can be formed from arginine with the help of N-carbamoyl putrescine and agmatine (Urano et al., 2003). This putrescine is also converted into spermine and spermidine by synthase enzyme. In saline conditions, out of these polyamines, putrescine is mainly accumulated. They interact with the membrane surface and with the help of their polyanionic nature, stabilize the membrane structure (Gill and Tuteja, 2010a,b). PAs also increase the membrane fluidity and act as nitrogen reserve so that plants use them after stress conditions are over. They also support in maintaining cellular pH and ionic balance. The main functions of polyamines are osmotic adjustment, scavenging hydroxyl radicals *via* modulating enzyme activities and ammonia detoxification (Kuznetsov et al., 2007; Mustafavi et al., 2018; Chen et al., 2019). It has been suggested that Spd level is a salt tolerance indicator (Li and He, 2012). Exogenously application of Spd, improved plant development *via* increased reactive oxygen metabolism and photosynthesis under salinity stress (Meng et al., 2015; Baniasadi et al., 2018). Various transgenic approaches are used to improve stress tolerance by expressing polyamine biosynthesis enzymes such as arginine decarboxylase (ADC), ornithine decarboxylase (ODC), spermidine synthase (SPDS) and S-adenosyl methionine decarboxylase (SAMDC; Gill and Tuteja, 2010a,b). Regulation of PAs becomes important in salt-stressed plants after confirmation of poor performance of transgenic plants mutated with PA synthesis genes (Urano et al., 2004; Marco et al., 2015). Up-regulated expression of Calvin-cycle-related genes mediated by PAs is responsible for the mitigation of detrimental effects of salinity in *Brassica napus* L. (ElSayed et al., 2022). The key enzymes involved in the Calvin cycle consist of *FBPase*, *PRKase*, *SBPase*, and Rubisco, which are important for CO_2_ fixation (Raines, 2003). Limitation in Rubisco activity has been deduced as one of the major constraints in the down-regulation of photosynthesis in salinity stress (Lu et al., 2009). However, exogenous application of Spd has altered the expression of *RbcL* and *RbcS* genes, which subsequently influences the Rubisco structure and function (Spreitzer, 2003). Polyamine oxidases (PAOs) are catabolic enzymes of PAs that are flavin adenine dinucleotide- dependent (Wu et al., 2022). Different PAOs, such as *AtPAO*_S_ and *ZmPAO*, have been identified in *Arabidopsis* and tobacco plants (Cona et al., 2006; Moschou et al., 2008). For instance, atpao5 in *Arabidopsis* stimulated the metabolic and transcriptional activities induced *via* salt stress (Zarza et al., 2017). Rice and wheat plants are identified with genes encoding proteins having PAO activity, suggesting an important function in salinity tolerance (Liu et al., 2014a,b; Xiong et al., 2017). Plant Polyamine oxidase (PAO) enzyme is responsible for H_2_O_2_ production during Put and Spd catabolism in plant tissues (Wang et al., 2019). Contrary to this, the *OsPAO3* gene has exhibited a positive effect in rice plants through the enhanced accumulation of polyamines, which is sufficient to eliminate overproduction of H_2_O_2_ and exclude Na^+^ (Liu et al., 2022).

## Salt overlay sensitive pathway

In terms of metabolic energy, plants use ions to balance water potential in tissues, unlike the use of carbohydrates or amino acids, which requires a significantly larger amount of energy. On the other hand, concentrations of ions should be maintained at an optimum level otherwise, it could be toxic to many cytosolic enzymes; therefore, compartmentalization of ions in the vacuoles becomes necessary (Binzel et al., 1998). As NaCl is the most common salt encountered by the plants during salinity stress, the salt overlay sensitive pathway is one of the main strategic approaches adopted by the plants. Plants perceive high Na^+^ concentration through downstream signaling of stress responses (Gong, 2021). The activation of Ca^2+^ channels always accompanies changes in ion concentrations and osmotic pressure. A putative osmosensor, OSCA1, is responsible for downstream Ca^2+^ signaling induced *via* osmotic stress (Yuan et al., 2014; Zhang et al., 2020). Similarly, the *Arabidopsis* mutant *moca1* (monocation-induced Ca^2+^) delivered a hypersensitive response to salt stress. These are identified as Na^+^-gated Ca^2+^ channels involved in the enhancement of Ca^2+^ concentration. GIPs (glycosyl inositol phosphorylceramide) are Na^+^ sensors encoded by MOCA1 to increase the influx of Ca^2+^ ions (Jiang et al., 2019). Cell-wall integrity is maintained by plasma membrane-positioned receptors-like kinases, FERONIA (FER), under salt stress (Feng et al., 2018). FER, along with BAK1 phosphorylate CNGC_S_ (cyclic nucleotide-gated ion channels) involved in Ca^2+^ signaling (Pan et al., 2019; Tian et al., 2019). Since salt stress triggers an overproduction of ROS, i.e., H_2_O_2_, a HPCA1 sensor located in the plasma membrane senses an increase in H_2_O_2_ concentration (Wu et al., 2020).

In a salt overlay sensitive pathway, SOS1, SOS2 and SOS3 are three genes commonly involved (Wu et al., 1996). During salinity stress, elevated Na^+^ stimulates a rise in cytosolic Ca^2+^ concentration, which interacts with Ca^2+^ binding protein SOS3, and further interaction with Serine/threonine kinase protein SOS2 mediates the efflux of Na^+^ from the cells through Na^+^/H^+^ antiporter (SOS1; Shi et al., 2003; Kim et al., 2007). SOS3 and SOS2 kinase complex directly phosphorylates SOS1. Furthermore, SOS1, SOS2 and SOS3 work together to provide resistance to salinity stress (Mahajan and Tuteja, 2005; Park et al., 2016). Furthermore, another member of SOS3 family, Calcineurin B-like (CBL10), forms a complex with SOS2, which is thought to regulate both the exclusion of Na^+^ ion through SOS1 and the compartmentalization of Na^+^ ion into the vacuole by activating Na^+^/H^+^ antiporter (NHX). A schematic representation of salt overlay sensitive signaling has been depicted in Figure 2.

## Salt stress signaling pathways regulates osmoprotectants accumulation

Plants perceive abiotic stress by activating signal-transduction pathways that allow them to adapt to even minor environmental changes. A wide array of complex transduction pathways are involved in sensing salt stress and generating a response during salinity stress. Pathways such as Salt overlay sensitive (SOS) pathway, phytohormone signaling, Calcium signaling, and Mitogen-Activated Protein Kinase (MAPK) network are involved in production and accumulation of osmolytes in regulating osmotic homeostasis during salinity stress (Roychoudhury and Banerjee, 2017).

The biosynthesis and accumulation of osmolytes is one of the important events in the activation of stress signaling pathways in plants during exposure to abiotic stress such as salt, heavy metals, cold, drought etc., that enables the plants to adapt quickly to changing environmental conditions. The SOS pathway is reported to be regulated by MAP Kinase pathways and the level of osmoprotectant GB also affects this pathway (Ashraf and Foolad, 2007). Similarly, abscisic acid signaling regulates proline accumulation in response to salinity stress as well as drought (Verslues and Bray, 2005).

In order to withstand constant stressful conditions, phytohormone mediating stress tolerance has been found to be crucial in plant response to salinity stress. Since phytohormones makes a great contribution is sensing salinity stress and adaption, nine plant hormones are commonly involved, which are divided into two groups: growth promoting hormones and stress response hormones (Yu et al., 2020). Auxins, gibberellins (GAs), cytokinins (CKs), brassinosteroids (BRs) and strigolactones (SLs) are growth-promoting hormones, while abscisic acid (ABA), ethylene, salicylic acid (SA) and jasmonic acid (JA) are stress response hormones. Among all, abscisic acid is most important in regulating salinity stress responses. Under stressful conditions, ABA accumulation takes place, which further activates kinase cascades and initiates stress defense reactions (Zhu, 2016). The Sucrose-nonfermenting-1-protein kinases 2 s (SnRK2s) are the main components in abscisic acid signaling pathways.

## Phytohormone signaling mediates osmoprotectants biosynthesis under salinity stress

**Figure 3:**
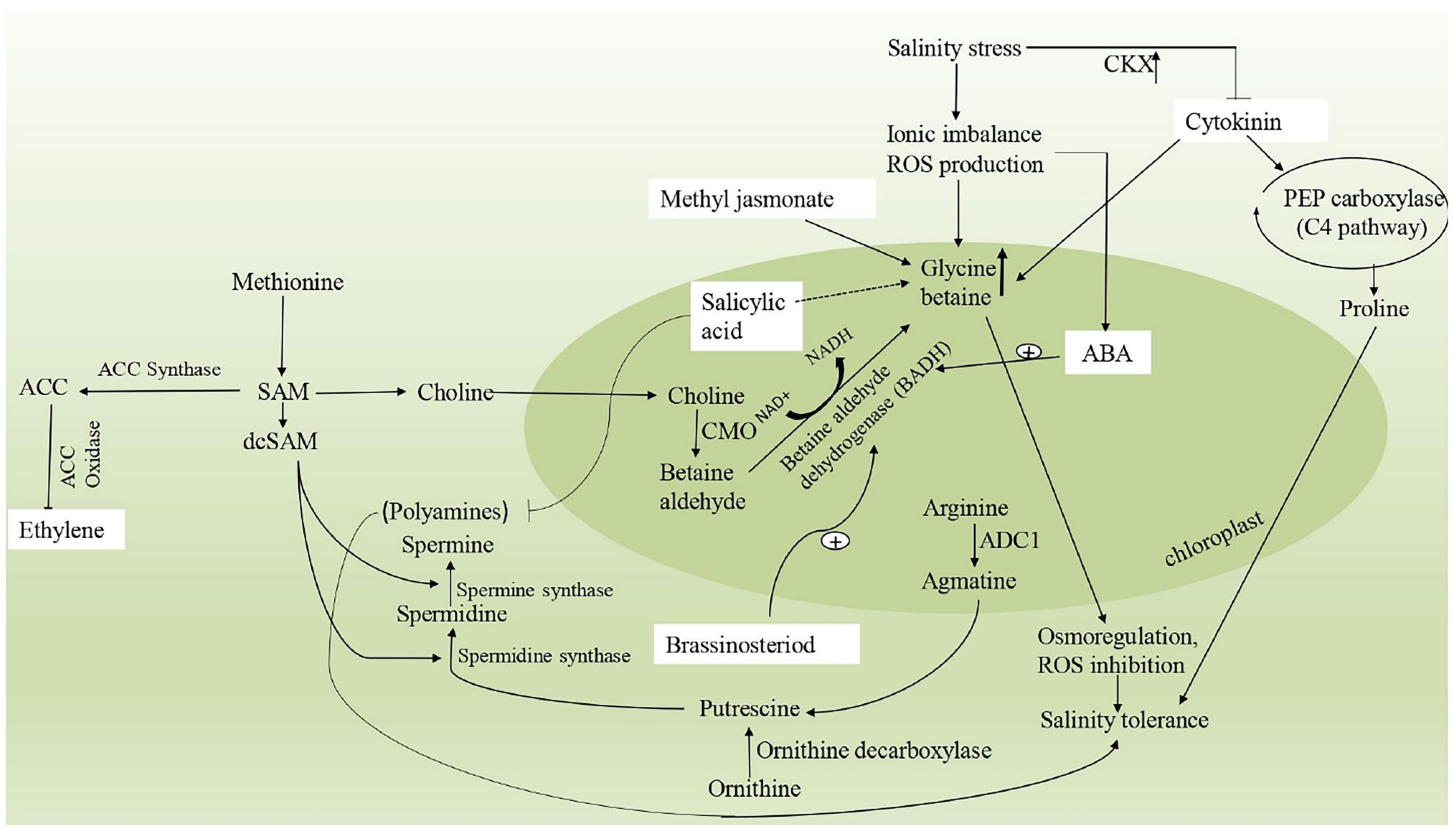
A schematic representation describing the interaction of different osmoprotectants with phytohormones under salinity stress. Glycine betaine and Ethylene are interlinked by a common pathway involving enzymes, BADH (betaine aldehyde dehydrogenase), CMO (choline monooxygenase), SAM (S-adenosylmethionine), ACC synthase and ACC oxidase. Polyaminebiosynthesis includes ornithine and arginine decarboxylation, catalyzed by ornithine decarboxylase (ODC) and arginine decarboxylase (ADC), respectively. Spermidine (Spd) is synthesized from putrescine *via* spermidine synthase (SPDS) with the addition of aminopropyl moiety donated by decarboxylated S-adenosylmethionine (dcSAM). Similarly, Spermine (Spm) is produced from spermidine (Spd) *via* spermine synthase (SPMS) with the same aminopropyl rendered by dcSAM. Cytokinins are deactivated by CKX1 (cytokinin oxidase/dehydrogenase) gene on exposure to high salinity stress.

The plant responds to salinity stress by the accumulation of compatible solutes as already discussed above. In order to alleviate osmotic stress, osmolytes further modulate the enhanced expression of genes involved in synthesis of plant hormones such as abscisic acid (ABA), cytokinins (CK), salicylic acid (SA), jasmonic acid (JA), auxins, polyamines, brassinosteroids (BRs) and gibberellins (GRs) (Fahad et al., 2015; Rao et al., 2016). These hormones has diverse role in modulating the signaling pathways during the emergence of salinity stress (Ali et al., 2020). Since, osmolytes and phytohormones have been elucidated to have a significant role under demanding environmental conditions; it is therefore becoming crucial to understand the regulation of osmolytes and phytohormones and further correlate the roles of the same. The interaction of phytohormones with osmoprotectants has been explained in Figure 3.

### Brassinosteroids

Many physiological processes in plants are controlled by brassinosteroid signaling during salt stress. BRL3, a member of the brassinosteroid (BR) receptor family, regulates biosynthesis of many key osmoprotectants (Fabregas et al., 2018). Soluble osmolytes sugars maintain osmotic homeostasis and helps in ROS scavenging (Parvaiz and Satyawati, 2008). Proline acts as a molecular chaperone by scavenging free radicals and also stabilizes redox reactions inside cytosol (Kumar et al., 2013). Applications of proline in addition to brassinosteroid (24-epibrassinolide) to cultivars of *Brassica juncea* that grow in saline environment minimize harmful effects of salinity and improves yield (Wani et al., 2019). GB is a type of compatible osmoprotectant, which never changes at neutral pH; hence highly water soluble but in the hydration sphere of proteins, is insoluble. So these sugar osmolytes withhold water and preserve the protein structure (Kurepin et al., 2017). Artificial supply of brassinosteroids provides salt stress tolerance to plants by enhancing GB secretion as illustrated in Figure 3. Exogenous application of 24-epibrassinolide to tolerant and sensitive varieties of *Pisum sativum* plants was reported in enhancing the GB content and further helps in mitigation of salinity stress (Shahid et al., 2014). In another study, Cd stressed *Pisum sativum* plants when subjected to 24-epibrassinolide it causes an additional increase in the level of GB, providing a stress tolerance ability to plants (Ahmad et al., 2018). Treatment of *Zea mays* L. seedlings with HBL (28-homobrassionolide) and EBL (epibrassinolide) resulted in the modulation of antioxidative enzyme activities and compatible osmolytes that accounts for osmotic adjustment during salt stress (Rattan et al., 2020). Hence, brassinosteroid has stress defending capacity by increasing osmolyte.

### Ethylene

Ethylene performs various physiological processes in the plant involving seed germination, plant development, senescence, fruit ripening, it has also been known to have a significant role in stress tolerance. It regulates abiotic stress by accumulating osmoprotectants (Iqbal et al., 2015). S-adenosyl methionine (SAM), the precursor for ethylene biosynthesis, is also a precursor for GB biosynthesis (Figure 3). Increasing GB and lower ethylene content (by inhibiting the activity of ACC synthase enzyme) in salinity stress enhances glutathione concentration (GSH) hence reducing oxidative stress (Khan et al., 2014). Proline along with ethylene gives salt tolerance capacity to *Brassica juncea* (Iqbal et al., 2015). Exogenous application of ethephon (ethylene source) with both doses of N and S on salt-stressed mustard plants increased the metabolism of proline, which is responsible for reducing oxidative stress (Jahan et al., 2021). The reason behind this fact is that increased nitrogen levels subsequently enhanced proline accumulation to provide salt tolerance (Iqbal et al., 2015). The biosynthesis of polyamines is also linked to ethylene production in terms of presence of precursor (S-adenosylmethionine, SAM; Petruzzelli et al., 2000). According to reports, ethylene and PAs are highly correlated in response to (ROS) production in the leaves of spring wheat seedlings under osmotic stress (Li et al., 2004). Increased PAs content, reduced ROS production, and ethylene synthesis were found in plants in response to stressful conditions.

### Cytokinins

Cytokinins are growth-promoting phytohormones and regulate plant growth and development (Pavlu et al., 2018). It promotes cell division in plant tissue culture and also regulates the cell cycle (Schaller et al., 2014). For osmolytes synthesis and accumulation in stress, ethylene cell signaling, MAPK plays a crucial function in plants (Shen et al., 2014). In abiotic stress, Cytokinin Oxidase/Dehydrogenase (CKX1) gene encodes for the enzyme that deactivates active cytokinins and increases the accumulation of GB content (Kathuria et al., 2009). Similarly, in *Physcomitrella patens*, overexpression of PpCKX1 lowers cytokinin levels and increases salt tolerance (Hyoung et al., 2019). These CKX-induced cytokinin-deficient plants are more valuable for researching the role of cytokinin than ipt mutants (Werner et al., 2003). The supply of cytokinin with NaCl gives signals that stimulate PEP carboxylase accumulation along with proline. Cytokinin activates PEP carboxylase that is responsible for increased proline levels in *M. crystallinum* (Thomas et al., 1992) and improved salinity stress tolerance (Figure 3). Cytokinins also regulate GB biosynthesis by modulating the GB pathway. In *Solanum lycopersicum*, the potential of Kinetin (Kn) and epibrassinolide (EML), either separately or in combination, was investigated under salinity stress. Results suggest that by enhancing the other physiological process, also leads to accumulation of GB through a crosstalk mechanism (Ahanger et al., 2020). Interestingly, several studies demonstrate that cytokinins and PAs govern various physiological and biochemical processes in plants, with a strong link between their levels, and operate as inter and intracellular messengers, regulating biotic and abiotic stresses (Galston, 1983; Wimalasekera and Scherer, 2009). However, the mechanism by which cytokinins influence the accumulation of PAs in plants is still unknown.

### Abscisic acid

Abscisic acid (ABA) maintains osmotic adjustment in salt-stressed plants by regulating physiological processes for osmolyte regulation (Karimi and Ershadi, 2015). At a molecular level, ABA controls synthetic pathways of osmolytes by acting as signaling molecules (Pal et al., 2018). It also increases proline synthesis and accumulation to protect plant cells from damaging effects (Karimi and Ershadi, 2015). This increased proline accumulation is due to an increase in the transcription of genes encoding essential enzymes in proline biosynthesis. In *Medicago truncatula*, it was reported that proline accumulation occurs under water deficit stress conditions controlled by ABA levels (Planchet et al., 2014). ABA increases the biosynthesis route of GB, resulting in increased accumulation of this osmolyte in plant cells, which aids in abiotic stress resistance (Zhang et al., 2012). Under abiotic stress after adding ABA to plant synthesis of GB level enhanced due to increased activity of GB biosynthetic enzyme betaine-aldehyde dehydrogenase (BADH; Yang et al., 2015; Figure 3). By analyzing drought-stressed plants after fluridone treatment, these researchers were able to confirm the significance of ABA in betaine-aldehyde dehydrogenase upregulation. In PAs production, ABA regulates critical transcriptional processes. For instance, gene transcript of arginine decarboxylase 2 (ADC2), spermidine synthase1 (SPDS1), and spermine synthase (SPMS) were found to be up-regulated under drought conditions (Alcázar et al., 2006). Similar trends were also reported in arginine decarboxylase (ADC) expression patterns in response to salinity stress (Urano et al., 2004). In plants, such as *Atriplex halimus, Oryza sativa, Phaseolus vulgaris* and *Zea mays*, the impact of ABA levels in response to salinity stress has also been reported (Liu et al., 2005; Ben Hassine et al., 2009; Shevyakova et al., 2013). ABA priming has been used to provide tolerance to abiotic stress such as drought, cold, or salt stress (Savvides et al., 2016). In addition, one-time ABA priming in *Vicia faba* grown under 50 mM salinity has been found to alleviate salt stress through alteration in gene expression patterns over time, maintaining the ionic and osmotic balance and increasing photosynthesis and growth (Sagervanshi et al., 2021).

### Jasmonic acid

In abiotic stress, jasmonic acid (JA) includes several plant reactions such as gene regulation, synthesis of particular proteins, and secondary metabolism. JA regulates the detrimental effects of environmental stress through a cascade of plant responses (Choudhary and Agrawal, 2014a,b; Choudhary et al., 2021). For example, JA increased the levels of non-enzymatic antioxidants such as proline, which has been reported in several studies (Anjum et al., 2011; Shan et al., 2015). Under salinity stress, methyl jasmonate greatly mitigated the adverse effects of salinity on soybean growth (Yoon et al., 2009). Exogenous jasmonates (JAs) supplement enhances plant growth because of their effect on metabolites. Application of JAs increases the accumulation of constituents of Krebs cycle and finally gives resistance to stressed plants (Sharma et al., 2016). In *Solanum lycopersicum*, the application of JAs increases the production of GB and thus enhance plant growth and development under salinity stress conditions (Ahmad et al., 2017, 2018; Figure 3). Salinity-induced peroxidation have been observed under exogenous application of methyl jasmonate in *Brassica napus* due to enhanced soluble sugar levels in leaves (Ahmadi et al., 2018). Furthermore, JA signaling have also been implicated in the production of PAs in fruit ripening, insect-pathogen tolerance, low-temperature injuries. Due to their primary role in activating essential antioxidant enzymes, Spd levels increased in barley genotypes and protected membranes from peroxidation (Bandurska et al., 2003). Similarly, under any form of stress, sugar level rises, JA has been demonstrated to enhance sugar content in a variety of crop plants, including *Triticum aestivum*, *Brassica napus*, *and Ipomea batata*, as well as improve overall plant performance under abiotic conditions (El-Khallal, 2001; Harpreet et al., 2013).

### Salicylic acid

Salicylic acid (SA), as a vital phytohormone, has multifaceted role in plant growth and developmental processes such as photosynthesis, mineral ion absorption and assimilation, antioxidant, and tolerance to stress (Choudhary and Agrawal, 2014a,b; Moravcová et al., 2018; Choudhary et al., 2021). The importance of SA in increasing resilience to environmental challenges has been well documented (Ahmad et al., 2011; Khan et al., 2015). During abiotic stress, the synthesis of osmolytes like GB, proline and sugar are influenced by SA (Misra and Misra, 2012; Jangra et al., 2022). The elevation in proline accumulation in salt-stress plants attributed to a stress tolerance mechanism (Misra and Saxena, 2009). In *Rauvolfia serpentina,* SA involves in enhancing proline production under salt stress and regulating cell turgor. It also mitigates salt stress in the seedling of *Torreya grandis* by accumulating proline. More proline concentration enhances the synthesis of stress protective proteins increasing stress tolerance (Li et al., 2014). SA enhances the accumulation of proline by regulating proline biosynthesis gene expression pattern. For instance, the exogenous application of SA mediates upregulation of important genes like P5CSA and P5CSB (encodes pyrroline-5-carboxylate synthase) and downregulation of PDH (encoding proline dehydrogenase; Lee et al., 2019).

SA improves the overall growth of the plant by influencing the concentration of GB (Misra and Misra, 2012; Farhadi and Ghassemi-Golezani, 2020). On the other hand, by applying exogenous SA and its analogs, scientists have determined its imperative role in ameliorating salt stress on various plants (Gharbi et al., 2017; Shaki et al., 2018). Recently, in *Vigna radiata* plants, supply of 0.5 mM SA enhanced the accumulation of GB by more than 40% with respect to control and thus minimizing the adverse of salinity (Syeed et al., 2021; Figure 3). On application of exogenous SA to salinity stressed *Zea mays*, SA increases the level of soluble sugars in this plant (Khodary, 2004). On the other hand, SA, prevents the accumulation of PAs under salt stress conditions (Palma et al., 2013).

## Conclusion and future prospects

Salt stress in crop plants is a potential threat for agricultural crop productivity and ultimately to food security under the current and futuristic Climate-changing scenarios, worldwide. The mechanism of salinity tolerance in plants is complex involving osmotic stress and ion toxicity causing a major loss of yield and quality. The role of phytohormones in regulating responses to adverse effects of salinity has been well documented however the studies related to phytohormone mediating osmolyte biosynthesis require more research insight to get a clear mechanism of crops tolerance. Therefore, in the present article we have summarized the roles of phytohormones in regulating osmolytes and further enhance our knowledge by explaining the cross talk at the physiological level on exposure to salinity. Apart from this, still, molecular dissection studies are required to unravel the mechanism behind the modulation of osmolytes. Among all phytohormones, the role of cytokinin remains contradictory, which needs to be focused. Various transgenic approaches have been elucidated in salt-sensitive plants that successfully impart salinity tolerance in plants. However, the deployment of novel approaches involving phytohormone engineering metabolism could be considered a method of choice to produce salt-resilient crops with higher yields.

## Author contributions

PS, SG, MS, BT, and SS: conceptualization, writing original draft, and writing—review and editing. NC: visualization and writing—review and editing. KC: conceptualization, visualization, supervision, and writing—review, and editing. All authors contributed to the article and approved the submitted version.

## Conflict of interest

The authors declare that the research was conducted in the absence of any commercial or financial relationships that could be construed as a potential conflict of interest.

## Publisher’s note

All claims expressed in this article are solely those of the authors and do not necessarily represent those of their affiliated organizations, or those of the publisher, the editors and the reviewers. Any product that may be evaluated in this article, or claim that may be made by its manufacturer, is not guaranteed or endorsed by the publisher.
